# Fine Needle Aspiration: An Atraumatic Method to Diagnose Head and Neck Masses

**DOI:** 10.5812/traumamon.10541

**Published:** 2013-10-13

**Authors:** Jamal Akhavan-Moghadam, Mahdi Afaaghi, Ali Reza Maleki, Amin Saburi

**Affiliations:** 1 Trauma Research Center, Baqiyatallah University of Medical Sciences, Tehran, IR Iran; 2 Department of Surgery, Baqiyatallah University of Medical Sciences, Tehran, IR Iran; 3 Health Research Center, Baqiyatallah University of Medical Sciences, Tehran, IR Iran; 4 Atherosclerosis and Coronary Artery Research Center, Birjand University of Medical Sciences, Birjand, IR Iran

**Keywords:** Diagnosis, Biopsy, Fine-Needle, Sensitivity and Specificity, Predictive Value of Tests

## Abstract

**Background:**

Patients presenting with a mass require tissue biopsy for histological diagnosis and treatment. Fine needle aspiration (FNA) is offered as an atraumatic, well tolerated, and inexpensive method for obtaining a biopsy from these lesions.

**Objectives:**

In this study we evaluated the accuracy of FNA as an atraumatic method among patients with nonthyroidal masses for diagnosis of neoplastic masses compared to open surgery.

**Patients and Methods:**

In a cross-sectional study, 65 patients with a head and neck masses (nonthyroidal) referred to us from 2004 to 2009. Those who had both FNA and open biopsy (the gold standard) were assessed for specificity, sensitivity, positive and negative predictive values of FNA in diagnoses.

**Results:**

Sixty-five cases with both definite diagnoses of open biopsy and FNA were assessed. The mean (± standard deviation) age of patients was 39.96 ± 19.69 years (range 10 to 82 years). Twenty-five (40.8%) subjects were categorized as malignant neoplasms, 16 (19.4%) as benign neoplasms, and 24 (39.8%) as non-neoplastic lesions. The sensitivity, specificity, positive and also negative predictive values of FNA in the diagnosis of neoplastic masses were 95%, 85%, 92.68%, and 91.66% respectively, and the diagnostic accuracy was 92.3%.

**Conclusions:**

It seems that FNA is a useful atraumatic diagnostic technique with a high diagnostic accuracy which can provide a highly sensitive diagnosis with low false positive diagnoses in patients with nonthyroidal masses.

## 1. Background

Many of serious and malignant disorders can initially present as a cervical mass; therefore, patients presenting with a head or neck mass need tissue biopsy to make a histological diagnosis and treatment plan. There have been developments in the diagnosis of head and neck masses in recent years. Many years ago when fine needle aspiration (FNA) was proposed, many studies were conducted to assess its accuracy rate (1). Most head and neck masses are a result of a hyperplastic process, benign or malignant tumor. FNA has been routinely recommended for diagnosis (2-4). Most of these studies have confirmed that FNA is a safe, reliable, well tolerated, and inexpensive method for diagnosis in patients with cervical masses including thyroid and nonthyroid masses (5). Due to the high rate of complications of open biopsy and surgery such as vascular complications (e.g. aneurysm), local spreading of the neoplasm, and cosmetic problems, surgeons sought a minimally invasive and atraumatic method for diagnosing tumoral masses (6). In addition, some patients are not suitable candidate for any surgical procedure even a small open biopsy due to their general condition (7). FNA cytology is a useful atraumatic technique for the first evaluation of cervical mass or nodule but its diagnostic accuracy and value in differentiation of neoplastic (including malignant or benign) and non-neoplastic masses is a debatable issue. The sensitivity of FNA in diagnosis of malignant mass ranges from 70% to 100% (3, 8). During recent years, FNA technique, devices including radiologic assisted FNA, and indications of this diagnostic procedure have improved. FNA is performed by physicians, pathologists, radiologists, general surgeons and otolaryngologists. 

## 2. Objectives

In this study we evaluated the diagnostic accuracy of FNA in patients with nonthyroidal masses in the head and neck.

## 3. Patients and Methods

### 3.1. Study Design and Participants

In a cross-sectional study 65 patients with head or neck masses (nonthyroid) referred to us from April 2004 to April 2009; they had both FNA and open biopsy (gold standard method) and were compared to assess specificity, sensitivity, positive and negative predictive values of FNA. 

### 3.2. Sampling

 Aspiration was performed by an expert pathologist using a 22-gauge needle and a 20 mL syringe. There was no major complication observed after aspiration. Two or more specimens were obtained each time FNA was performed, and smears were directly prepared for cytology; after fixing via alcohol, smears were stained using Papanicolaou stain. Cytology of specimens obtained by FNA was followed by open biopsy during surgery, and the permanent diagnosis in every patient was made by histological study. 

### 3.3. Ethics and Statistical Analysis

As a routine in this hospital, all subjects signed a form of informed consent before sampling. The study protocol was approved by the ethical and scientific committee of our university. SPSS version 16 was used for statistical analysis. Mean ± standard deviation (SD), t-test, ANOVA, and Chi-square tests were used for the analysis, and P < 0.05 was considered as statistically significant. Also the sensitivity, specificity, positive predicting value, and negative predicting value were estimated using a diagnostic chart (Table 1). 

**Table 1.** Chart Used for Estimating the Test Characteristics.

FNA Findings ^a^	Diagnosis by Open Biopsy	
	Abnormal, No. (%)	Normal, No. (%)
**Test outcome abnormal**	True Positive, 38 (67.8)	False positive, 3 (4.61)
**Test outcome normal**	False negative, 2 (3.07)	True negative, 22 (33.8)

^a^Abbreviation: FNA, fine needle aspiration

Statistical measures were estimated as follows; Sensitivity = True Positive/Abnormal in gold standard, Specificity = True Negative/ Normal in gold standard, Positive predictive value = True Positive/Observed Test Outcome Positive, Negative predictive value = True Negative/Observed Test Outcome Negative, Diagnostic Accuracy = True positive plus True negative/ All subjects.

## 4. Results

Sixty-five cases with both definite diagnoses of FNA and open biopsy were assessed. The mean (± SD) age of patients was 39.96 ± 19.69 years (from 10 to 82 years). Twenty-nine cases (44.6%) were females and 36 (55.4%) males. Regarding the results of open surgical biopsy; 25 (40.8%) subjects were categorized as malignant, 16 (19.4%) cases as benign, and 24 (39.8%) cases as non-neoplastic lesions (Table 2). 

**Table 2.** Diagnosed Specimens

Diagnosis	No.	Subgroup, %	Total, %
**Malignant neoplasms**			
Basal cell carcinoma	1	4	1.5
Adenocarcinoma	2	8	3
Acinic cell carcinoma	1	4	1.5
Clear cell carcinoma	1	4	1.5
Hodgkin lymphoma	5	20	7.5
Non-Hodgkin lymphoma	2	8	3
Lymphoma	3	12	4.5
Metastatic carcinoma	10	40	15.4
**Benign neoplasms**			
Pleomorphic adenoma	13	81.25	20
Warthin tumor	1	6.25	1.5
Lipoma	2	12.5	3
**Non-neoplastic lesions**			
Inflammatory process	2	8.33	3
Granulomatous	3	12.5	4.5
Branchial cyst	3	12.5	4.5
Sialoadenitis	1	4.16	1.5
Thyroglossal cyst	4	16.66	6
Dermoid cyst	1	4.16	1.5
Globulomaxillary cyst	1	4.16	1.5
Reactive lymphadenitis	9	37.4	13.8

The most common type of malignant neoplastic mass diagnosed was metastatic carcinoma (40%), and also the most common masses among benign neoplastic and benign masses were pleomorphic adenoma and reactive lymphadenitis (81.25% and 37.4%, respectively). The mean age between the three groups was significantly different, and post-HOC test demonstrated that the differences between the mean age of two groups of neoplastic (benign neoplastic = 43.62 ± 16.42 and malignant = 50.97 ± 22.31) and non-neoplastic (benign = 31.79 ± 15.58) were statistically significant (P < 0.001). Six (12.24%) of 65 subjects were diagnosed incorrectly (Table 3). 

**Table 3.** Diagnosis of Specimens Which Were Incorrect

FNA Report ^a^	Open Biopsy
Metastatic carcinoma	Reactive lymphadenitis
Pleomorphic adenoma	Basal cell carcinoma
Lymphoma	Reactive lymphadenitis
Inflammatory process	Metastatic carcinoma
Reactive lymphadenitis	Lymphoma

^a^Abbreviation: FNA, fine needle aspiration

There were 38 true positive, 22 true negative, 3 (4.61%) false positive and 2 (3.07%) false negative diagnoses by FNA for neoplastic masses. Therefore, the specificity, sensitivity, positive and also negative predictive values of FNA in the diagnosis of neoplastic mass were estimated as 85%, 95%, 92.68%, and 91.66%, respectively, and also the diagnostic accuracy was estimated as 92.3%.

## 5. Discussion

In this study, FNA cytology was performed in neck masses of the cervical region. Regarding the result, it seems that FNA is a useful atraumatic and minimally invasive technique with high diagnostic accuracy which can provide a highly sensitive diagnosis with low false positive and also low false negative diagnosis in patients with nonthyroid head or neck mass. In previous reports, FNA was effectively used for staging diseases and also designing a safe, less invasive and atraumatic management plan. FNA can prevent unnecessary, costly investigation in patients with a cervical mass (9). The proportion of nondiagnostic procedures was 8.9% of all procedures(146 cases) which was similar to other previous studies (10). Also, Mehrotra et al. reviewed the literature and reported a 5.0-43.1% unsatisfactory rate in the initial assessment of FNA cytology which was similar to our results (11). In a similar study of thyroid and nonthyroid masses, Saatian et al., found the sensitivity and specificity of FNA lower than our findings which can be due to inclusion of thyroid masses in their investigation (8). Also, our false negative proportion (3.07%) is much less than the Saatian et al. (26%) and QueHee et al. (39%) studies. On the other hand the proportion of false positive results for the diagnosis of malignant mass in our study was about 4% which is higher than Morgan et al. findings (0%) (12).The differences in the results of various studies can be due to the technique of aspiration. Regarding the prevalence of malignant lesions in head and neck masses, the initial use of FNA cytology for the confirmation or exclusion of this diagnosis is mandatory. Some studies confirm that the accuracy of FNA can depend on the pathologic type of mass (13). Also, Biopsy can enhance the diagnostic accuracy of FNA. Amedee et al., in a review article demonstrated that FNA followed by biopsy has a high overall accuracy of 87% for malignant mass, 95% for benign mass, and 95% for all head and neck ones (14). In contrary, Addams-Williams et al., showed that FNA was highly sensitive for diagnosis of malignant lumps, but less good at confirming a benign lump (15). Some techniques such as electron microscopy, flow cytometry, and immunohistochemistry were offered to increase the accuracy rate of FNA particularly for masses based on lymph nodes (16). Also, ultrasound-guided FNA can reduce nondiagnostic rate (17). Also, we found that patients with neck mass who were characterized as a malignant neoplasm were older than those diagnosed as nonmalignant (consistent with previous reports) (18).On the other hand, in the present study the diagnosis of tubercular lymphadenitis was established in 4.5% which was much less than the values obtained in other studies in neighboring countries (2, 13). This difference can be due to the lower prevalence of tuberculosis in our country compared to our neighbors. Also, it was approved that the accuracy of FNA depends on operators experiences. Jandu et al., reported that the accuracy of FNA in head and neck mass was 91% when was performed by junior staff, and 100% when was performed by a consultant (19). Moreover, we reviewed similar studies to evaluate the accuracy of FNA in the diagnosis of head and (or) neck masses, and the range of diagnostic accuracy was between 56% and 100%, the range of sensitivity 55% - 100%, and the range of specificity was 59% - 100% (Table 4). FNB is newer than FNA, and is used in more advance stage or for more doubtful findings. Also, FNB is usually recommended when the result of FNA is not valid or suspicious. As seen in the literature review, the sensitivity, specificity and accuracy of FNA were different due to many different factors. The country and technology which is used in that country can affect the diagnostic accuracy. Also, experience can influence the result. This difference in diagnostic accuracy of FNA in various countries can be due to the experience of physicians and technology. Another reason for a wide difference in the FNA results is the location of the mass. Locating in a deeper and central site can decrease the accessibility for taking a biopsy. The target diagnosis can change the diagnosing accuracy. For example, in Table 4, the accuracy of FNA is higher in cases which underwent biopsy for diagnosing malignancy. The age (adult or child) can change the sensitivity and specificity. An important factor is the year of study. As was said before, developing technology usually improves the accuracy of all tests. Moreover, our literature review showed that FNA is a minimally invasive procedure for the diagnosis of neck masses, although recently additional methods such as ultrasonography increase the diagnostic value (20). Also, FNA as an atraumatic method can help to design an effective surgical plan (21) in addition to identifying the tumor characteristics (22). 

**Table 4.** Literature on Accuracy, Sensitivity, and Specificity of FNA^a^

	Name	Year	Country	No.	Age, y	Location	Accuracy, %	Sensitivity, %	Specificity, %	Mass
**1**	Our Study	2012	Iran	65	Above 10	Neck	92.3	95	85	Malignancy
**2**	Santos et al. (23) ^b^	2011	Brazil	50	Above 10	Oral, Head & neck	58.8	75	96	Benign
**3**	Saatian et al. (8)	2011	Iran	100	42.6	Neck	79	72	87	Metastatic malignancy
**4**	Carr et al. (24)	2010	UK	NA	Adults	Head and neck	87	85	91	Lymph nodes
**5**	Kuvezdic et al. (25)	2010	Croatia	100	All	Head and neck	NA	90	88	Lymphoma
**6**	Moatamed et al. (26)	2009	US	92	All	Head and neck	NA	76	100	Cyst
**7**	Addams-Williams et al. (15)	2009	UK	625	Above3	Non-Thyroid neck lumps	88 - 66	92	90	Malignant
**8**	Hirachand (3)	2009	Nepal	130	3-85	Head and neck	NA	100	100	Metastatic carcinoma
**9**	Carrillo et al. (27) ^b^	2009	Mexico	NA	All	Parotid mass	NA	92	98	Malignancy
**10**	Anne et al. (28) ^b^	2008	US	71	8.4	Head and neck	92	100	85	Malignancy
**11**	Tandon et al. (29)	2008	UK	Review	All	Head and neck	95.1	89.5	98.5	Malignancy
**12**	Hernandez et al. (30)	2008	Colombia	46	All	Parotid mass	48	54	90	Cancer
**13**	Arabi et al. (31) ^c^	2008	US	31	All	Head and neck	NA	97	100	Malignancy
**14**	Gonzalez et al.(32)	2008	Spain	172	All	Head and neck	NA	95.8	98.11	Malignancy
**15**	Howlett et al. (17)	2007	UK	143	Adult	Head and neck	NA	89	57	Overall
**16**	Martinek et al. (33)	2004	Czech	245	All	Thyroid nodules	86	90	85	Malignancy
**17**	Morgan et al. (12)	2003	Australia	253	All	Thyroid nodules	67.2	55	73.7	Malignancy
**18**	El Hag (13)	2003	SA	225	All	Head and neck	NA	95	96	Cancer
**19**	Contucci et al. (34) ^b^	2003	Italy	146	All	Parotid mass	94	57.2	100	Malignancy
**20**	Tilak et al. (35)	2002	India	55	NA	Head and neck	92.73	90.91	93.18	Overall
**21**	Tilac et al. (35)	2002	India	55	All	Head and neck	92.7	90.9	93.1	Overall
**22**	Arda et al. (9) ^b^	2001	Turkey	46	child	Thyroid	59	60	59	Thyroid nodules
**23**	Quehee et al. (10)	2001	Australia	169	All	Parotid mass	56	57	100	Malignancy
**24**	Amedee et al. (14) ^b^	2001	U.S	Review	adult	Head and neck	95	NA	NA	Overall
**25**	Mehrvarz and Sangsari (36)	2001	Iran	95	adult	Thyroid nodules	NA	78	92	Overall
**26**	Bakhos et al. (37)	2000	US	625	All	Thyroid nodules	87	93	96	Malignancy
**27**	Jandu et al. (19)	1999	UK	95	All	Head and neck	91-100	90	97	Malignancy
**28**	Al-Khafaji et al. (38)	1998	US	154	All	Parotid mass	84	82	86	Malignancy

^a^Abbreviations: FNA, fine needle aspiration; NA, not available; SA, Saudi Arabia; y, year

^b^FNA findings confirmed by fine needle biopsy

^c^FNA during the operation
